# Exploring the relationship between total serum calcium and melanoma development: a cross-sectional study

**DOI:** 10.3389/fnut.2024.1461818

**Published:** 2024-12-23

**Authors:** Qiaochu Zhou, Wei Wang, Jinhui Wang, Changchang Li, Jianle Ji

**Affiliations:** Wenzhou Hospital of Integrated Traditional Chinese and Western Medicine, Wenzhou, China

**Keywords:** melanoma, NHANES, total serum calcium, LASSO, cross-sectional study

## Abstract

**Background:**

Melanoma is the fourth leading cause of cancer-related death worldwide. The continuous exploration and reporting of risk factors of melanoma is important for standardizing and reducing the incidence of the disease. Calcium signaling is a promising therapeutic target for melanoma; however, the relationship between total serum calcium levels and melanoma development remains unclear.

**Methods:**

In this study, we included patients with melanoma from the National Health and Nutrition Examination Survey (NHANES) database from 2003 to 2006 and from 2009 to 2016. The baseline clinical characteristics of the participants were analyzed using the chi-square and rank-sum tests. Subsequently, a fitted model was constructed to evaluate the relationship between total serum calcium levels and melanoma development. The performance of total serum calcium levels and covariates in predicting the risk of melanoma was assessed based on ROC curves. Finally, LASSO regression analysis was performed using the “glmnet” R package to identify clinical characteristics associated with melanoma.

**Results:**

A total of 13,432 participants were included in this study. Age, race, household poverty-to-income ratio, response of the skin to sunlight after a certain period of non-exposure, wearing long-sleeved shirts, frequency of sunscreen use, and arthritis were significantly correlated with the development of melanoma. The *p*-values of total serum calcium levels in three fitted models were < 0.05, and the OR values were < 1. According to the ROC curves, the AUC values of models 2 and 3 were 0.728 and 0.766, respectively, indicating that the combination of total serum calcium levels and covariates showed better performance in predicting the occurrence of melanoma. Furthermore, LASSO regression analysis revealed seven clinical characteristics significantly associated with melanoma.

**Conclusion:**

This study revealed a relationship between total serum calcium levels and melanoma development. Total serum calcium levels combined with phenotypic and clinical characteristics were found to be more effective in predicting the occurrence of melanoma. Therefore, the relationship between total serum calcium levels and melanoma development warrants further investigation in prospective cohort studies.

## 1 Introduction

Melanoma is a life-threatening malignant skin tumor that originates from melanocytes. In addition to affecting the skin, melanocytes can form pigments in the eyes, ears, leptomeninges, gastrointestinal tract, oral mucous membranes, genital mucous membranes, and sinonasal mucous membranes. Based on clinical and pathological features, nine subtypes or pathways of melanoma have been identified (1). Melanoma is the fourth leading cause of cancer-related death worldwide (2), representing a serious public health concern. In 2017, the age-adjusted incidence of melanoma in the United States was 22.7 cases per 100,000 people, which was projected to increase by 5.8% by 2021 (3). In a study, a combinatorial cluster analysis of melanoma images revealed five morphological subtypes of melanoma as follows: typical, nevus-like, non-pigmented/non-melanoma skin cancer-like, seborrheic keratosis-like, and freckle-like/lentigo maligna-like (4). Cutaneous melanoma accounts for >90% of all melanoma cases in White populations (5). Although melanoma accounts for only 3% of all skin cancer cases, 65% of all skin cancer-related deaths are caused by melanoma (6). The mainstay of treatment for melanoma is surgical removal of the primary tumor, whereas other treatment options are recommended based on staging. The time interval between diagnosis and treatment is a determinant of survival in patients with melanoma (7). With the progress and development of society, the living environments and habits of individuals are also changing. Therefore, to reduce the incidence of melanoma, its risk factors should be continuously explored and updated and actively applied to the screening of high-risk patients.

Mitochondria primarily perform many functions related to cellular metabolism and homogenous stability. The outer mitochondrial membrane plays an important role in calcium signaling between mitochondria and the endoplasmic reticulum. Endoplasmic reticulum stress in the tumor microenvironment is a common driver of tumor progression. The uncontrolled proliferation of malignant tumor cells leads to the production of an unfavorable microenvironment characterized by high metabolic demands, hypoxia, nutrient deficiencies, and acidosis, all of which in turn disrupt calcium homeostasis. These harmful conditions alter the protein-folding capacity of the endoplasmic reticulum in cancer cells and tumor-infiltrating immune cells. This alteration promotes the accumulation of misfolded or unfolded proteins within the endoplasmic reticulum, leading to endoplasmic reticulum stress (8). Hypoxia may have different effects on cancer cells and tumor-infiltrating immune cells throughout cancer development. It induces the release of exosomes via monocarboxylate transporter isoform 1 (MCT1) and CD147 in a calcium-dependent manner (9). The planar cell polarity (PCP) pathway is divided into Wnt and calcium pathways (10). The Wnt signaling pathway influences cellular phenotypic changes, promotes treatment resistance, and regulates the immune microenvironment in melanoma (11). The pathway that identified pathways associated with cancer progression included the calcium signaling pathway (12). Calcium ions are involved in regulating cell motility, gene transcription, muscle contraction, and exocytosis. Intracellular Ca^2+^ signaling plays a crucial role in modulating various cellular functions. Moreover, the spatial and temporal coordination of Ca^2+^ signals within cells is as important as stimulation by chemoattractants extracellularly. During cell migration, Ca^2+^ signaling is involved in directional sensing, cytoskeleton redistribution, and relocation of focal adhesion (13). Disruption of calcium homeostasis has been identified as a key factor involved in various processes related to cancer development, including cancer cell growth, migration, and invasion (14).

Altered mitochondrial function in melanoma cells can directly affect melanoma development, whereas altered mitochondrial function in immune cells can indirectly affect melanoma development (15). Intracellular calcium concentration ([Ca^2+^]i), calcium channels on the cell membrane, calcium signaling-related proteins (S100 family proteins, E-Ca^2+^, E-calmodulin, and calpain), cytosolic calcium channels, and the Wnt/Ca^2+^ pathway have been associated with the development and progression of melanoma. According to a cross-sectional study, an increase in albumin-corrected serum calcium levels may serve as a marker of disease progression in cutaneous malignant melanoma (16). Calcium signaling affects the melanoma microenvironment, including immune cells, the extracellular matrix (ECM), the vascular network, and the chemical and physical environments. A study reveals that under conditions of extracellular Ca^2+^ presence, mouse melanoma cells show a reduction in both actin cables and tubulin networks of mouse melanoma cells. This change in the morphology of melanoma cells is attributed to an abnormally excessive uptake of Ca^2+^ by the cells from the culture medium, resulting in a distortion of the cell shape (17). The ionophore application in a basal medium containing Ca^2+^ initiated a sustained influx, and Ca^2+^ is a potential down-regulator of alpha2beta1-integrin ligation of A2058 human melanoma cell chemotaxis to type IV collagen (18).

Other ion channels, such as sodium and potassium channels, are also involved in the calcium signaling pathway in melanoma. Calcium signaling is a promising therapeutic target for melanoma, and its dysregulation may serve as a predictive marker for the disease (19). Although serum calcium is reported to be involved in the development and progression of melanoma, its specific molecular mechanisms remain elusive. Moreover, observational studies investigating the relationship between total serum calcium levels and the risk of melanoma are lacking at present.

This study aimed to investigate the role of total serum calcium in melanoma. The National Health and Nutrition Examination Survey (NHANES) is a continuous survey of the health and nutrition of the US population. In this study, we used serum calcium data from the NHANES database to investigate the relationship between total serum calcium levels and the risk of melanoma.

## 2 Materials and methods

### 2.1 Study population

Data reported between 2003 and 2006 and between 2009 and 2016 were retrieved from the NHANES database.^1^ Participants lacking data on melanoma and covariates and having a history of other cancers were excluded. Eventually, a total of 13,432 participants were selected for subsequent analysis (control = 13,393; case = 39).

### 2.2 Disease definition

Participants who had responded “Yes” to MCQ220 of the Medical Conditions Questionnaire (MCQ), “Have you ever been told by a doctor or health professional that you have a malignant tumor?” and “Melanoma” to MCQ230, “What kind of cancer?” were identified as patients with melanoma and included in the case group. Participants who had responded “No” to question MCQ220 were included in the control group.

### 2.3 Covariates

The following covariates were included in this study: age (years, mean ± SD) (RIDAGEYR), race (Mexican American, Other Hispanic, Non-Hispanic White, Non-Hispanic Black, and Other Races) (RIDRETH1), sex (male and female) (RIAGENDR), education level (9th grade or lower, 9–11th grade, high school graduate, some college, and college graduate or above) (DMDEDUC2), household poverty-to-income ratio (mean ± SD) (INDFMPIR), body mass index (mean ± SD) (BMXBMI), drinking status (yes and no) (ALQ101), cigarette smoking (yes and no) (SMQ020), response of the skin to sunlight after a certain period of non-exposure (get a severe sunburn with blisters, a severe sunburn for a few days with peeling, mildly burned with some tanning, turning darker without a sunburn, nothing would happen in half an hour, and other reactions) (DED031), staying in shadows (always, most of the time, sometimes, rarely, and never) (DEQ034A), wearing long-sleeved shirts (always, most of the time, sometimes, rarely, and never) (DEQ034C), frequency of sunscreen use (always, most of the time, sometimes, rarely, and never) (DEQ034D), sunburn (yes and no) (DEQ038G), high cholesterol levels (yes and no) (BPQ080), arthritis (yes and no) (MCQ160A), chronic bronchitis (yes and no) (MCQ160K), diabetes mellitus (yes and no) (DIQ010), high blood pressure (yes and no) (BPQ020), and serum total calcium levels (mean ± SD) (LBXSCA). Baseline statistics were shown in Table 1.

**Table 1:** Baseline characteristics of the participants.

	level	Overall	0	1	p
n		13432	13393	39	
Drink (%)	No	3389 (25.2)	3382 (25.3)	7 (17.9)	0.388
	Yes	10043 (74.8)	10011 (74.7)	32 (82.1)	
BMI (mean (SD))		29.40 (7.31)	29.40 (7.30)	29.03 (7.85)	0.749
High_cholesterol (%)	No	9658 (71.9)	9634 (71.9)	24 (61.5)	0.206
	Yes	3774 (28.1)	3759 (28.1)	15 (38.5)	
High_blood_pressure (%)	No	10099 (75.2)	10072 (75.2)	27 (69.2)	0.499
	Yes	3333 (24.8)	3321 (24.8)	12 (30.8)	
Age (mean (SD))		39.72 (11.11)	39.70 (11.11)	47.64 (9.96)	<0.001
Race (%)	Mexican American	1969 (14.7)	1969 (14.7)	0 ( 0.0)	<0.001
	Non-Hispanic Black	2911 (21.7)	2908 (21.7)	3 ( 7.7)	
	Non-Hispanic White	5861 (43.6)	5827 (43.5)	34 (87.2)	
	Other Hispanic	1103 ( 8.2)	1102 ( 8.2)	1 ( 2.6)	
	Other Race	1588 (11.8)	1587 (11.8)	1 ( 2.6)	
Gender (%)	Female	6941 (51.7)	6917 (51.6)	24 (61.5)	0.283
	Male	6491 (48.3)	6476 (48.4)	15 (38.5)	
Education_level (%)	9-11th Grade	1553 (11.6)	1550 (11.6)	3 ( 7.7)	0.366
	College Graduate or above	3811 (28.4)	3798 (28.4)	13 (33.3)	
	High School Graduate	2843 (21.2)	2839 (21.2)	4 (10.3)	
	Less Than 9th Grade	752 ( 5.6)	750 ( 5.6)	2 ( 5.1)	
	Some College	4473 (33.3)	4456 (33.3)	17 (43.6)	
PIR (mean (SD))		2.69 (1.67)	2.69 (1.67)	3.34 (1.65)	0.015
Skin_reaction (%)	A severe sunburn for a few days with peeling	1105 ( 8.2)	1096 ( 8.2)	9 (23.1)	0.001
	Get a severe sunburn with blisters	270 ( 2.0)	269 ( 2.0)	1 ( 2.6)	
	Mildly burned with some tanning	3439 (25.6)	3423 (25.6)	16 (41.0)	
	Nothing would happen in half an hour	5366 (39.9)	5358 (40.0)	8 (20.5)	
	Other	119 ( 0.9)	119 ( 0.9)	0 ( 0.0)	
	Turning darker without a sunburn	3133 (23.3)	3128 (23.4)	5 (12.8)	
Stay_in_the_shade (%)	always	1310 ( 9.8)	1307 ( 9.8)	3 ( 7.7)	0.136
	Most of the time	3398 (25.3)	3381 (25.2)	17 (43.6)	
	Never?	1260 ( 9.4)	1257 ( 9.4)	3 ( 7.7)	
	rarely, or	2136 (15.9)	2131 (15.9)	5 (12.8)	
	sometimes	5328 (39.7)	5317 (39.7)	11 (28.2)	
Long_sleeved_shirt (%)	always	619 ( 4.6)	617 ( 4.6)	2 ( 5.1)	0.034
	Most of the time	760 ( 5.7)	754 ( 5.6)	6 (15.4)	
	Never?	6584 (49.0)	6572 (49.1)	12 (30.8)	
	rarely, or	2735 (20.4)	2724 (20.3)	11 (28.2)	
	sometimes	2734 (20.4)	2726 (20.4)	8 (20.5)	
Sunscreen (%)	always	1465 (10.9)	1456 (10.9)	9 (23.1)	0.009
	Most of the time	1828 (13.6)	1818 (13.6)	10 (25.6)	
	Never?	5391 (40.1)	5383 (40.2)	8 (20.5)	
	rarely, or	1977 (14.7)	1972 (14.7)	5 (12.8)	
	sometimes	2771 (20.6)	2764 (20.6)	7 (17.9)	
Sunburn (%)	No	8192 (61.0)	8173 (61.0)	19 (48.7)	0.159
	Yes	5240 (39.0)	5220 (39.0)	20 (51.3)	
Diabetes (%)	No	12371 (92.1)	12337 (92.1)	34 (87.2)	0.399
	Yes	1061 ( 7.9)	1056 ( 7.9)	5 (12.8)	
Arthritis (%)	No	11278 (84.0)	11254 (84.0)	24 (61.5)	<0.001
	Yes	2154 (16.0)	2139 (16.0)	15 (38.5)	
chronic_bronchitis (%)	No	12799 (95.3)	12764 (95.3)	35 (89.7)	0.208
	Yes	633 ( 4.7)	629 ( 4.7)	4 (10.3)	
Smoke (%)	No	7924 (59.0)	7904 (59.0)	20 (51.3)	0.414
	Yes	5508 (41.0)	5489 (41.0)	19 (48.7)	
Total_Calcium (mean (SD))		9.40 (0.36)	9.40 (0.36)	9.30 (0.40)	0.104

### 2.4 Statistical analysis

The chi-square test (for categorical variables) and the rank-sum test (for continuous variables) were used to compare clinical characteristics between the control and case groups, and baseline statistical tables were used to present the results. The relationship between total serum calcium levels and the risk of melanoma was estimated by constructing fitted models using the “survey” package in R (version 4.4-1) (20).

This process included weighting, first, we calculated a new variable, preweight, by dividing WTMEC2YR (the weight of the sample over a two-year cycle) by six. Subsequently, using the svydesign function, we defined a sampling design object. This object contained the following core components: the primary sampling unit (PSU) was specified through ids = ~SDMVPSU, where SDMVPSU was the key variable for sample grouping; the stratification variable was identified through strata = ~SDMVSTRA, which represented the basis for stratification in the sampling process; the weights = ~ preweight specifies the weight variable, here the preweight variable that had been calculated earlier is used; by setting nest = TRUE, a weighted sampling design object called stamoch lead was finally obtained, which integrated the information of stratification, weight and clustering and ensured that the subsequent analyses can accurately reflect the NHANES sampling design characteristics. Model 1 incorporated the risk of melanoma and total serum calcium levels. Model 2 was adjusted for age, sex, household poverty-to-income ratio, education level, and body mass index based on model 1. Model 3 further incorporated drinking status, smoking status, response of the skin to sunlight after non-exposure, staying in shadows, wearing long-sleeved shirts, frequency of sunscreen use, sunburn, high cholesterol levels, arthritis, chronic bronchitis, diabetes, and high blood pressure. The “pROC” package in R (version 1.18.0, PMID:21414208) was used to plot the receiver operating characteristic (ROC) curves of the three models and calculate the area under the curve (AUC) values. The “glmnet” package in R (version 4.1-4, PMID: 20808728) was used to perform LASSO regression analysis to identify clinical characteristics that were significantly associated with the risk of melanoma. A *p*-value of <0.05 indicated statistical significance. Sample information and number of covariates are in Supplementary Table S1.

## 3 Results

### 3.1 Baseline statistics

A total of 13,432 participants (control = 13,393; case = 39) from the NHANES database were included in this study. The baseline clinical characteristics of these participants are shown in Table 1. Several covariates were compared between the control and case (melanoma) groups. The results showed that age, race, household poverty-to-income ratio, response of the skin to sunlight after non-exposure, wearing long-sleeved shirts, frequency of sunscreen use, and arthritis were significantly associated with the development of melanoma.

### 3.2 Association of total serum calcium levels with melanoma risk

**Figure 1:**
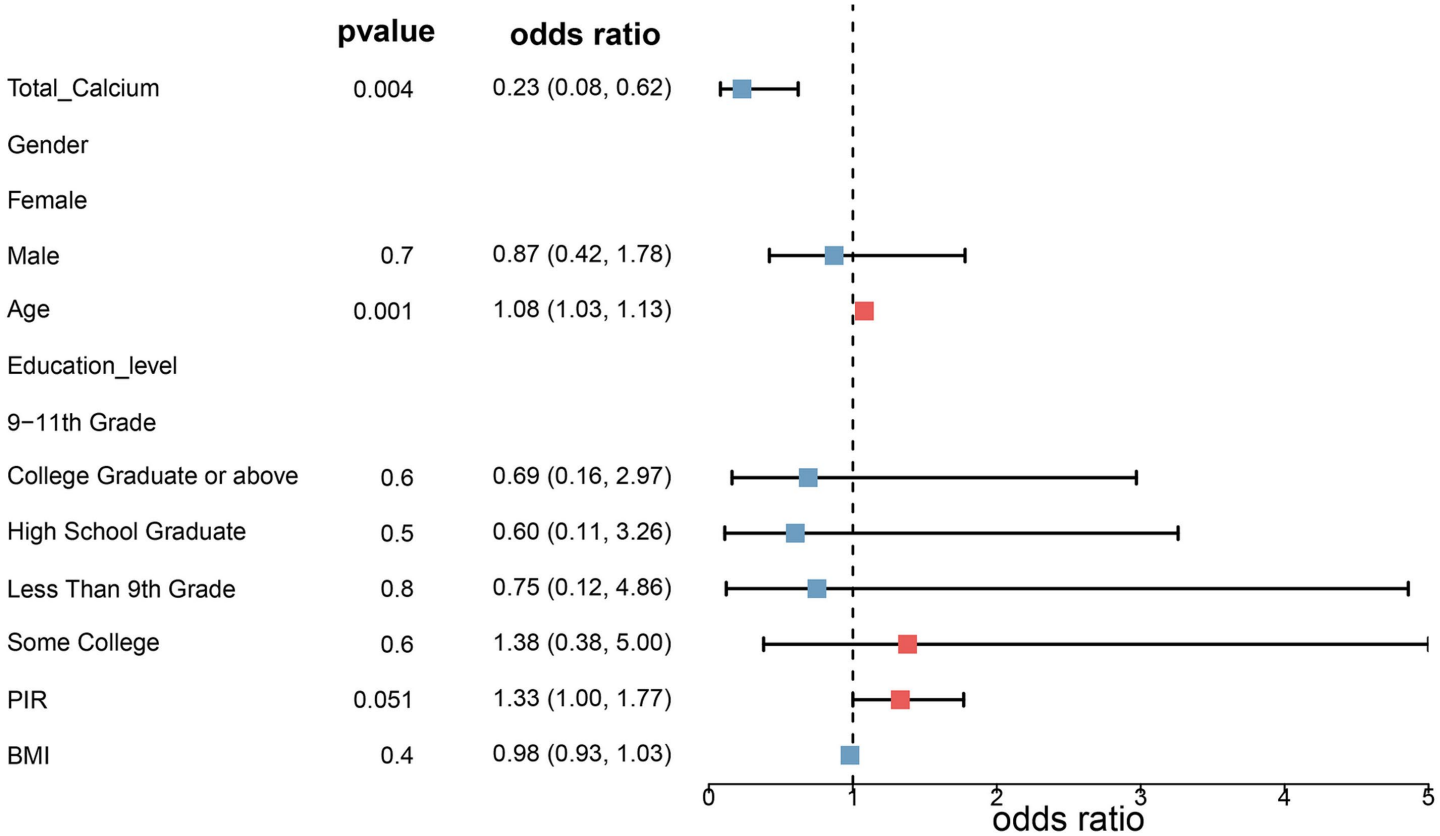
Risk ratios in model 2. Model 2 was adjusted for age, sex, household poverty-to-income ratio, education level, and body mass index based on model 1, and model 3 further incorporated drinking status, smoking status, response of the skin to sunlight after non-exposure, staying in shadows, wearing long-sleeved shirts, frequency of sunscreen use, sunburn, high cholesterol levels, arthritis, chronic bronchitis, diabetes, and high blood pressure.

**Figure 2:**
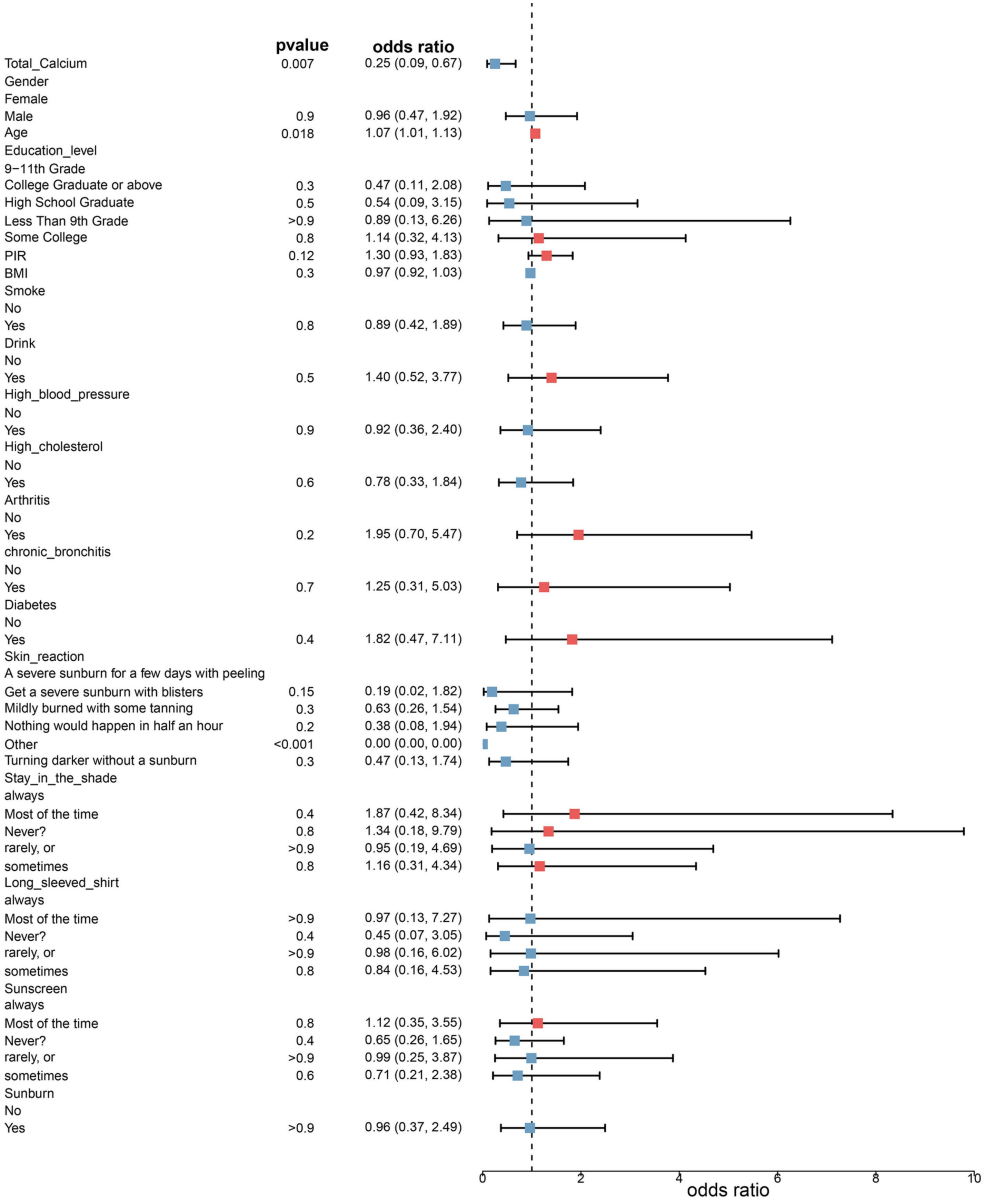
Risk ratios in model 3.

Three fitted models were constructed to assess the relationship between total serum calcium levels and the risk of melanoma. The risk ratios of the three models are shown in Table 2 and Figures 1, 2. In all three models, the *p*-values of total serum calcium levels were < 0.05 and the OR values were < 1, which indicated that total serum calcium had a significant effect on the development of melanoma and was negatively correlated with the risk of melanoma. In addition, age had a significant effect on the development of melanoma.

**Table 2:** Risk ratios in the fitted models.

**Characteristic**	**OR** ^1^	**95% CI** ^1^	**p-value**
Total_Calcium	0.22	0.08, 0.65	0.007

^1OR = Odds Ratio, CI = Confidence Interval^

### 3.3 ROC curves

**Figure 3:**
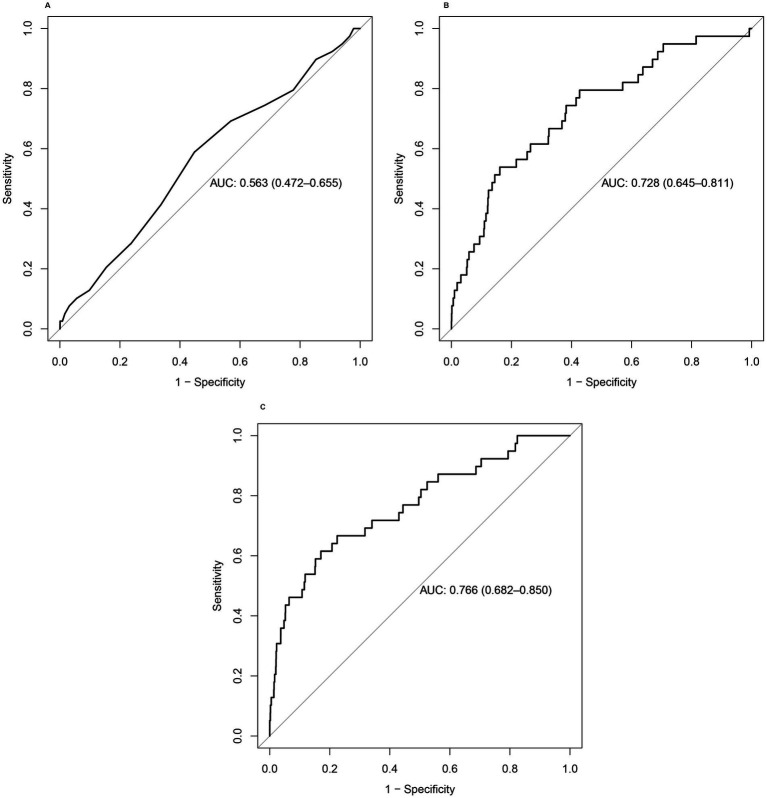
**(A–C)** ROC curves of the three models. The combination of total serum calcium levels with other covariates showed better performance in predicting the occurrence of melanoma.

The ROC curve is considered the most important tool for evaluating the performance of medical diagnostic tests and predictive models. The closer the AUC value to 1, the stronger the predictive ability of the test or the model. The AUC values of models 2 and 3 were 0.728 and 0.766, respectively (Figure 3). These results indicated that total serum calcium levels combined with some other covariates had a superior ability to predict the occurrence of melanoma.

### 3.4 LASSO regression analysis

**Figure 4:**
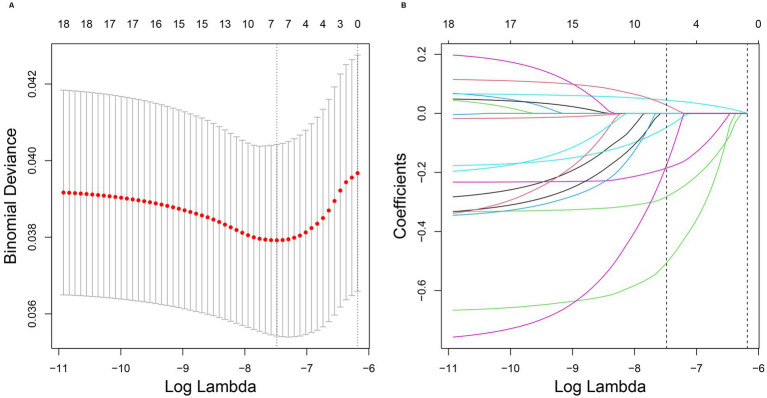
**(A,B)** Results of LASSO regression analysis. LASSO regression analysis correlation plot. The image on the left shows the plot of the penalty term parameter, with the horizontal coordinate representing the log(lambda) value and the vertical coordinate representing the degree of freedom, which represents the cross-validation error. In the actual analysis, it is hoped that the cross-validation of the error of the smallest position, in the figure, the left dashed position is the cross-validation of the smallest error, according to the position (lambda.min) to determine the topmost cross coordinate log(Lambda), the top shows the number of traits, find the optimal log(Lambda) value, find the corresponding trait and its coefficient in the right figure. In the image on the right, the horizontal coordinate represents log(lambda), whereas the vertical coordinate represents the coefficient of the trait (different variables with *λ*-penalization).

Clinical characteristics significantly associated with melanoma were identified using LASSO regression analysis. The LASSO model with the smallest error (lambda.min = 0.00056) was considered optimal (Figure 4). According to this model, age, household poverty-to-income ratio, response of the skin to sunlight after non-exposure, wearing long-sleeved shirts, frequency of sunscreen use, arthritis, and serum total calcium levels were significantly associated with the development of melanoma.

## 4 Discussion

In this study, we evaluated the relationship between total serum calcium levels and the risk of melanoma using data from the NHANES database. The clinical data of 13,432 participants (control = 13,393; case = 39) reported between 2003 and 2006 and between 2009 and 2016 were retrieved. Data on melanoma, total serum calcium levels, and some other covariates were used for summary analysis and model fitting. The relationship between each clinical characteristic and the risk of melanoma was analyzed using the chi-square (categorical variables) and rank-sum (continuous variables) tests. The results showed that age, race, household poverty-to-income ratio, response of the skin to sunlight after non-exposure, wearing long-sleeved shirts, frequency of sunscreen use, and arthritis were significantly associated with the occurrence of melanoma (*p* < 0.05). Subsequently, three fitted models were constructed to assess the relationship between total serum calcium levels and the risk of melanoma. In all three models, the *p*-values of total serum calcium levels were < 0.05 and the OR values were < 1. These results indicated that total serum calcium levels had a significant effect on the risk of melanoma and the two factors were negatively correlated. Among the other covariates, age was found to have a significant effect on the risk of melanoma. The AUC values of models 2 and 3 were 0.728 and 0.766, respectively, indicating that the combination of total serum calcium levels with some other covariates had a superior ability to predict the occurrence of melanoma. In addition, we reviewed previous studies and found similar results. One study showed that pathophysiological abnormalities associated with serum calcium levels appeared to be related to the accelerated development of arterial stiffness with age (21). There was also a study that concluded female gender was an independent favourable prognostic factor for melanoma survival (22). Cancer has various paraneoplastic effects, including hypercalcemia. A cross-sectional study of 644 patients with malignant melanoma showed that albumin-corrected calcium levels were positively associated with the stage of the disease. This finding suggests that albumin-corrected serum calcium levels can indicate melanoma progression before clinical signs are evident (16). Another study proposed a model demonstrating that metastatic melanoma leads to reprogramming of organ function through the formation of platelet-activating factor from long-chain polyunsaturated phosphatidylcholine under oxidative conditions and through the systemic induction of intracellular calcium mobilization. Calcium mobilization in platelets can alter the levels of disease markers therein (23). An increase or decrease in extracellular Ca^2+^ concentration significantly affected B16F10 melanoma cell motility, and the critical role of resting Ca^2+^ influx in driving the migration of B16F10 melanoma cells is emphasised (24). A decrease in serum albumin levels may increase the “free” fraction of serum calcium; however, parathyroid-related protein (PTHrP), the major cause of malignancy-induced hypercalcemia, is more likely to contribute to the increase in serum calcium levels (25). Melanoma cell growth and invasion are inversely controlled by Orai1 and STIM2 in a dynamic manner. Melanoma cells express high levels of Orai1 and STIM2, both of which control store-operated Ca^2+^ entry (26). Silencing Orai1/STIM2 or decreasing extracellular Ca^2+^ levels can decrease intracellular Ca^2+^ levels.

In this study, the combination of total serum calcium levels with some other covariates showed better performance in predicting the occurrence of melanoma. LASSO regression analysis revealed seven clinical characteristics that were significantly associated with the development of melanoma, namely, age, household poverty-to-income ratio, response of the skin to sunlight after non-exposure, wearing long-sleeved shirts, frequency of sunscreen use, arthritis, and total serum calcium levels. According to the three fitted models, age, in addition to total serum calcium levels, had a significant effect on the development of melanoma. In a study based on the Centers for Disease Control Wide-Ranging Online Data for Epidemiologic Research (CDC WONDER) database, participants aged > 65 years had a higher mortality rate (27). Consistently, a meta-analysis revealed that age exceeding 50 years was one of the prognostic factors associated with disease-specific survival after surgery (28). These findings are consistent with those of the present study. In younger adults with sun-exposed skin with minimal solar elastosis, superficial spreading melanomas frequently develop on the trunk or back, which are often associated with precursor nevi, whereas lentigo maligna-like melanomas usually develop on the head. In addition to protective clothing, hats, and eyewear, sunscreen plays a crucial role in preventing UV radiation from penetrating the skin (5). With regard to the relationship between sun-protective behaviors and a family history of melanoma, a study based on data from NHANES 2003–2004 showed that a larger proportion of individuals with a known family history of melanoma preferred sunscreen to other sun-protective behaviors (29). An analysis of 1999–2018 NHANES data with serum carotenoids associated with sunburn severity and cancer risk found a positive association between sunburn severity and cancer risk (30). The melanoma-associated clinical characteristics identified in this study are consistent with the abovementioned characteristics. Th9 cells exhibit both positive and negative relationships with melanoma and can trigger inflammatory responses in autoimmune diseases, such as rheumatoid arthritis (31). Compared with the general population, patients with treated rheumatoid arthritis are at a higher risk of developing melanoma. Therapeutic breakthroughs might have mitigated disease activity or on the contrary by impairing antitumoral immune response (32). In previous studies, attempts have been made to identify a single clinical feature as a risk factor for melanoma. However, in this study, we found that the total serum calcium level on its own is not suitable as a diagnostic marker for melanoma and that combining it with phenotypic and other clinical characteristics is necessary to predict the risk of melanoma more effectively. To ensure the validity of the results, we used appropriate weights for confounder adjustment during the analysis. Overall, the findings of this study have important implications for future research.

However, this study has some limitations that should be acknowledged. Although we obtained a large and nationally representative population cohort from the NHANES database, the number of cases in our study was relatively small and due to the cross-sectional nature of the present study, the causal relationship between total serum calcium levels and melanoma could not be established. The findings suggest that total serum calcium levels combined with phenotypic and clinical traits were more effective in predicting the occurrence of melanoma. However, given that the NHANES database contains self-reported data, recall bias cannot be ruled out, and the recall bias may affect our ability to make accurate judgements about the relationships between variables, leaving a degree of uncertainty in the conclusions we draw. Therefore, in the future, we will expand the time frame of data collection to include more participants in our study, using mechanistic analyses to determine causality. We plan to validate our self-reported data using methods such as repeated measures and cross-validation with other data sources to improve the reliability of self-reported data. In addition, we will further support our findings with *in vitro* experiments. We will continue to monitor research in this direction. Gene expression profiling approaches have been used for risk stratification, either independently or in combination with staging parameters. Circulating tumor DNA is also gaining significance as a tool for predicting the risk of early-stage melanoma (23). Serum calcium is an inexpensive laboratory test that is routinely ordered. Therefore, prospective studies are warranted to validate the clinical utility of total serum calcium levels in predicting melanoma progression and investigate the common mechanisms underlying the regulation of serum total calcium levels and melanoma development.

## 5 Conclusion

In conclusion, this study revealed a relationship between total serum calcium levels and the risk of melanoma, providing a theoretical basis for the clinical detection of melanoma based on total serum calcium levels. However, when used independently, the total serum calcium level is not suitable as a diagnostic marker for melanoma. Therefore, it should be combined with phenotypic and other clinical characteristics to accurately predict the occurrence of melanoma. In addition, the common mechanisms involved in the regulation of total serum calcium levels and melanoma development warrant further investigation.

## Data Availability

The original contributions presented in the study are included in the article/Supplementary material, further inquiries can be directed to the corresponding author.

## Footnotes

^1^ https://wwwn.cdc.gov/nchs/nhanes/search/
